# Antisense Oligonucleotide Therapy for Amyotrophic Lateral Sclerosis (ALS): An Umbrella Review

**DOI:** 10.7759/cureus.93140

**Published:** 2025-09-24

**Authors:** Edward Jeong, Dan Li

**Affiliations:** 1 College of Medicine, Choate Rosemary Hall, Farmington, USA; 2 General Medicine, Massachusetts General Hospital, Boston, USA

**Keywords:** als treatment, antisense oligonucleotides, antisense oligonucleotide therapy, familial amyotrophic lateral sclerosis, neurodegenerative disease

## Abstract

Amyotrophic lateral sclerosis, also known as ALS or Lou Gehrig's disease, is a fatal neurodegenerative disease prominent in the elderly population. To this point, no completely effective treatments have been procured; however, antisense oligonucleotide therapies, or ASOs, are a promising venue. In order to investigate the efficacy of ASOs in the treatment of ALS by targeting specific genetic mutations, we conducted an umbrella review utilizing keywords such as “ALS” and “ASO” in the PubMed database, excluding sources published more than 10 years ago for relevance. Results revealed that of multiple tentative ASO treatments, for multiple specific gene mutations, only one, Tofersen, was approved for the wider population. The main cause of failure was an inability to meet efficacy endpoints, resulting in the discontinuation of the product. Tofersen is able to treat mutations in the SOD1 gene, but not any others. While initially discouraging, the production of ASOs is a relatively new and advanced process, and slow progress is expected. However, there remains the problem of identifying and treating the much more prevalent sporadic ALS, which is much more common compared to familial ALS.

## Introduction and background

Amyotrophic lateral sclerosis (ALS) is a fatal neurodegenerative disorder affecting approximately 0.25% of the population, typically in adults aged 55 and older [1]. It primarily impacts motor neurons, leading to muscle atrophy, respiratory failure, and ultimately, death. Most patients present with limb weakness, dysarthria, and spasticity, and the disease generally progresses within two to five years of onset [2]. In its final stage, ALS often results in respiratory failure due to the widespread loss of muscle control. The course of the disease is unpredictable and ultimately fatal.

Although no effective treatments have been identified so far, many are under investigation. Among the most promising are oligonucleotide-based therapies. Oligonucleotides are short DNA or RNA molecules that, when used in ALS treatment, target neurotoxic mutant genes that harm the nervous system [3,4]. This approach is relatively new but has been extensively studied due to its potential to treat various neurodegenerative diseases.

Despite this promise, oligonucleotide therapy remains imperfect. Many questions remain about its effectiveness in treating ALS. Nevertheless, it stands out as the most hopeful therapeutic strategy for several neurodegenerative conditions. This review aims to objectively evaluate the effects and possibilities of oligonucleotide therapy as a potential treatment for ALS and assess its potential for treatment. We describe its side effects, delivery methods, and effectiveness in comparison with other treatment options.

This paper presents a comprehensive umbrella review of current evidence on oligonucleotide therapy for ALS. An umbrella review is a synthesis of findings from multiple systematic reviews and meta-analyses. In this review, we examine the therapeutic mechanisms, clinical outcomes, safety profiles, and implementation challenges of ASOs. 

## Review

Methods

Research Question

How has oligonucleotide therapy been used as a treatment for ALS?

*Research Methodology* 

To refine the scope of our investigation, we derived sub-questions from the main research question: 1. What is oligonucleotide therapy, and why is it being used for ALS?; 2. At what stage has this therapy been applied in ALS treatment?; 3. How does oligonucleotide therapy compare with other therapies in terms of efficacy?

Pubmed Search Strategy

We conducted a systematic search using PubMed with search terms aligned with our research focus. The final search strategy was as follows: (("Amyotrophic Lateral Sclerosis"(MeSH Terms) OR "Amyotrophic Lateral Sclerosis"(Title/Abstract)) AND ("Oligonucleotides"(MeSH Terms) OR "oligonucleotide*"(Title/Abstract))) AND ((meta-analysis(Filter) OR review(Filter) OR systematicreview(Filter)) AND (2015:2024(pdat))).

Screening Protocol 

Two rounds of screening were conducted. First, we screened titles and abstracts, followed by full-text reviews of the remaining manuscripts retrieved through our search. 

Inclusion Criteria

We focused on studies involving populations diagnosed with ALS, including all ages and geographic locations. To ensure relevance with recent advancements, we limited our review to articles published within the last decade (2015-2024). The primary concepts were antisense oligonucleotide (ASO) therapy and ALS. All included sources were obtained through PubMed.

Flow Chart/Data Extraction

We used the Preferred Reporting Items for Systematic Reviews and Meta-Analyses (PRISMA) flow chart to illustrate the study selection process-ranging from identification, duplicate removal, screening, and eligibility assessment to final inclusion. We also assessed study quality using the Joanna Briggs Institute's critical appraisal tools. All included articles were MEDLINE-indexed to ensure reliability. A charting template was developed to facilitate systematic data extraction.

Results

PRISMA Screening Process

**Figure 1:**
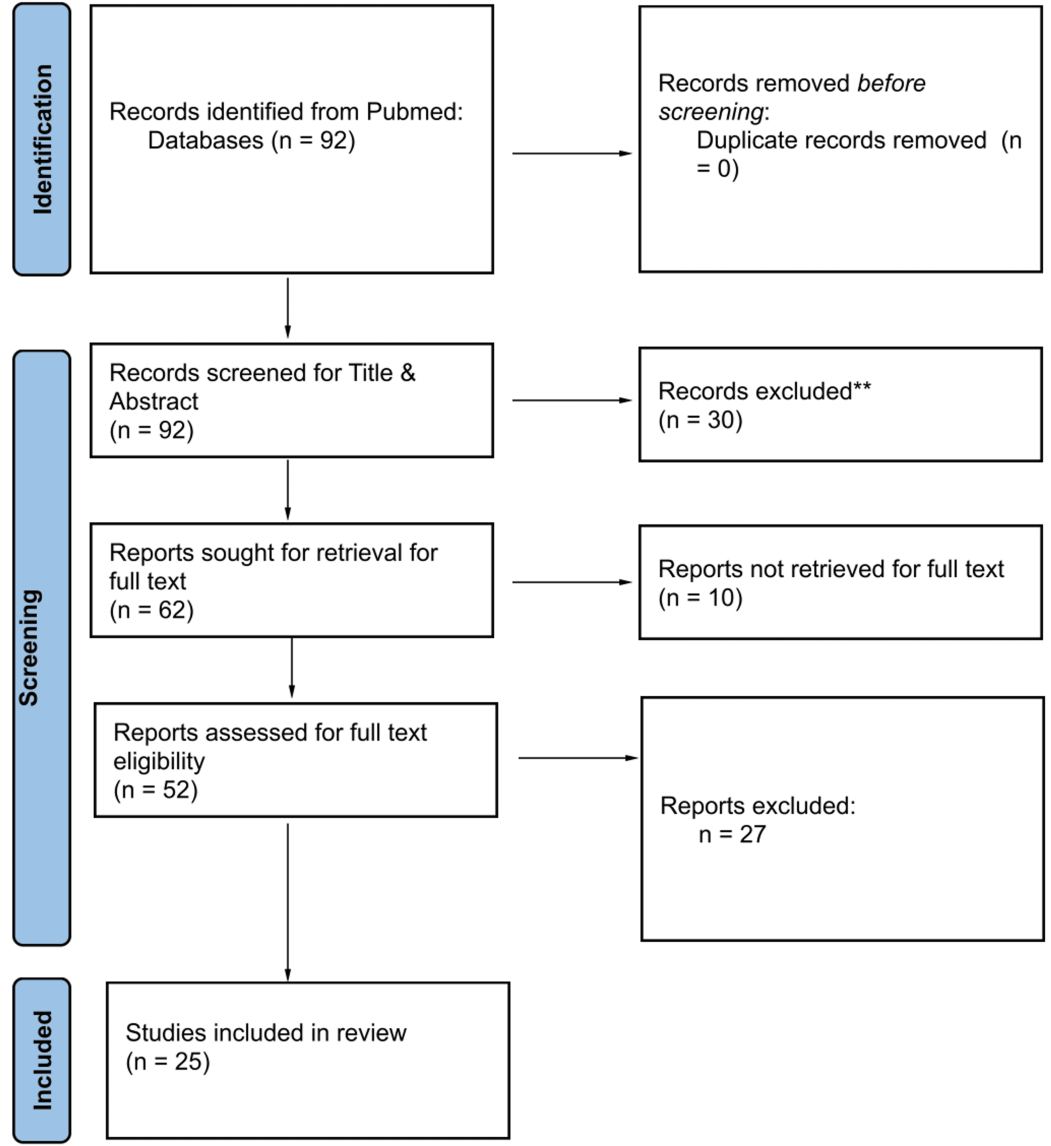
PRISMA flow chart detailing numeric values for screening process. PRISMA: Preferred Reporting Items for Systematic Reviews and Meta-Analyses.

A total of 92 studies were identified using the predefined search terms. After title and abstract screening, 30 studies were excluded due to irrelevance. An additional 27 were excluded during full-text screening for not meeting inclusion criteria, such as intervention specificity or the absence of outcome data. Ten full-text articles could not be retrieved despite multiple access attempts. Ultimately, 25 studies were included in this review (Figure 1). 

*Paper Characteristics* 

The selected studies, published between 2015 and 2024, investigated the efficacy of various antisense oligonucleotide (ASO) therapies targeting ALS-related gene mutations. Most were Phase I or III clinical trials, primarily focusing on genes such as silence superoxide dismutase 1 (SOD1), C9orf72, fused in sarcoma (FUS), TAR DNA binding protein (TARDBP), and Ataxin-2 (ATXN2). While the studies originated from diverse geographic regions, the location of origin did not influence inclusion (Table 1). 

**Table 1:** Comparative analysis of included studies. Table Credits: Edward Jeong.

Authors	Title	ASOs/causative gene referenced	Sample size
Suzuki et al. 2022 [1]	Genetics of amyotrophic lateral sclerosis: seeking therapeutic targets in the era of gene therapy	SOD1, fused in sarcoma (FUS), C9orf72	327
Meyer 2021 [2]	Amyotrophe Lateralsklerose (ALS)–Diagnose, Verlauf und neue Behandlungsoptionen	SOD1, C9orf72, TARDBP, FUS	21
Bennett et al. 2019 [3]	Antisense oligonucleotide therapies for neurodegenerative diseases	SOD1, C9orf72	137
Al Dera et al. 2024 [4]	Molecular mechanisms and antisense oligonucleotide therapies of familial amyotrophic lateral sclerosis	FUS	30
Amado et al. 2021 [5]	Gene therapy for ALS: a review	SOD1, C9orf72, ATXN2, FUS	168
Everett et al. 2024 [6]	Tofersen for SOD1 ALS	Tofersen	96
Fang et al. 2022 [7]	Gene therapy in amyotrophic lateral sclerosis	TARDBP	149
Mathis et al. 2018 [8]	RNA-targeted therapies and amyotrophic lateral sclerosis	SOD1, C9orf72, ATXN2, and TARDBP	77
Bagyinszky et al. 2023 [9]	Studies of genetic and proteomic risk factors of amyotrophic lateral sclerosis inspire biomarker development and gene therapy	SOD1, C9ORF72, TARDBP, FUS	274
Abati et al. 2020 [10]	Silence superoxide dismutase 1 (SOD1): a promising therapeutic target for amyotrophic lateral sclerosis (ALS)	SOD1	125
Boros et al. 2022 [11]	Antisense oligonucleotides for the study and treatment of ALS	SOD1, C9orf72, FUS, ATXN2	104
Saini et al. 2023 [12]	Breaking barriers with tofersen: enhancing therapeutic opportunities in amyotrophic lateral sclerosis	Tofersen	49
Sever et al. 2022 [13]	Comprehensive research on past and future therapeutic strategies devoted to treatment of amyotrophic lateral sclerosis	SOD1	211
Al Shaer et al. 2024 [14]	2023 FDA TIDES (peptides and oligonucleotides) harvest	Tofersen	64
Oliveira Santos et al. 202 [15]	Profiling tofersen as a treatment of superoxide dismutase 1 amyotrophic lateral sclerosis	Tofersen	43
Meijboom et al. 2022 [16]	Approaches to gene modulation therapy for ALS	SOD1, TDP-43	180
Cappella et al. 2019 [17]	Gene therapy for ALS-a perspective	SOD1, C9orf72	147
Cappella et al. 2020 [18]	Beyond the traditional clinical trials for amyotrophic lateral sclerosis and the future impact of gene therapy	SOD1, C9orf72, FUS	106
Wurster et al. 2018 [19]	Antisense oligonucleotides in neurological disorders	SOD1, C9orf72	172
Chamakioti et al. 2022 [20]	Advanced gene-targeting therapies for motor neuron diseases and muscular dystrophies	BIIB105 (TDP-43), WVE-004(C9orf72)	212
Wang et al. 2023 [21]	Recent progress of the genetics of amyotrophic lateral sclerosis and challenges of gene therapy	NEK1, CCNF, ANXA11, TIA1, KIF5A	112
Ito 2021 [22]	Promise of nucleic acid therapeutics for amyotrophic lateral sclerosis	SOD1, C9orf72, TDP43	75
Van Daele et al. 2024 [23]	The sense of antisense therapies in ALS	SOD1, C9orf72, FUS, ATXN2	74
Cantara et al. 2024 [24]	Antisense oligonucleotides (ASOs) in motor neuron diseases: a road to cure in light and shade	c9orf72, FUS, TDP-43	133
Ly et al. 2018 [25]	Emerging antisense oligonucleotide and viral therapies for amyotrophic lateral sclerosis	SOD1, C9orf72, TDP43	53

Risk of Bias Assessment

Using the Cochrane Risk of Bias 2.0 tool, each trial was assessed across five domains. The Tofersen Phase III trial was considered to have a low risk of bias due to rigorous randomization and blinding protocols. In contrast, trials for BIIB078 and WVE-004 showed a high risk due to the lack of significant clinical endpoints and incomplete outcome reporting. Case studies, such as those involving Jacifusen and Afinersen, presented a serious risk of bias due to their single-patient design and the absence of control groups. The BIIB105 trial had an unclear risk due to insufficient methodological details. 

As part of our screening criteria, we also applied the Joanna Briggs Institute (JBI) critical appraisal tools for assessing methodological quality. Each paper was evaluated based on the relevant JBI checklist for its study type. If a paper did not meet at least 80% of the required components outlined in the JBI assessment, it was excluded from the final analysis to ensure methodological rigor and minimize bias across included studies.

Oligonucleotide Therapy

Antisense oligonucleotides (ASOs) are short, single-stranded sequences of synthetic nucleotides designed to bind to specific mRNA transcripts. Once bound, they induce degradation of the target mRNA via the RNase H enzyme, thereby reducing the production of the corresponding protein [5]. This targeted mechanism offers a high degree of specificity, making ASOs particularly appealing for addressing diseases caused by known gene mutations.

The therapeutic appeal of ASOs lies in their ability to selectively silence mutated genes without affecting the wild-type counterparts. After an mRNA is cleaved by RNase H, the enzyme is free to bind and degrade additional mRNA molecules, leading to a sustained decrease in the expression of the pathogenic protein [6]. This repeatable mechanism amplifies the therapeutic effect of a single ASO molecule.

In recent years, ASOs have gained significant attention as potential treatments for various neurodegenerative diseases, including amyotrophic lateral sclerosis (ALS) and spinal muscular atrophy (SMA) [5]. A notable milestone was the FDA's approval of nusinersen in 2016 for the treatment of SMA, which demonstrated the clinical viability of ASO-based therapies. This success has bolstered efforts to develop ASOs targeting ALS and other neurological disorders.

Although still a relatively new class of therapeutics, ASOs represent a promising and rapidly evolving strategy for addressing gene-specific pathologies, particularly in conditions like ALS, where precise gene silencing could halt or slow disease progression [5].

Role in the Treatment of Amyotrophic Lateral Sclerosis (ALS)

Amyotrophic lateral sclerosis (ALS) is associated with a wide array of genetic mutations, with over a hundred implicated genes identified to date. Among the most commonly mutated are SOD1, C9ORF72, TARDBP, ATXN2, and FUS, each playing a significant role in familial ALS (fALS) pathogenesis [5,7,8]. For instance, mutations in SOD1, the first gene linked to fALS, account for approximately 15-30% of familial cases [9-11]. Similarly, the C9ORF72 gene, which features a hexanucleotide repeat expansion, is found in over 30% of European fALS patients but is much rarer in Asian populations, with a prevalence of less than 3%.

TARDBP and FUS mutations also vary by region. TARDBP mutations are present in 4.2% of familial ALS cases in Europe and 1.5% in Asia, while FUS mutations are more prevalent in Asia (6.4%) than in Europe (2.8%) [9]. These genes are rarely associated with sporadic ALS (sALS), which makes up the majority of ALS diagnoses.

ASOs aim to reduce the expression of these mutant gene products to mitigate cellular toxicity, slow neuronal degeneration, and delay disease progression. This gene-specific strategy is particularly promising for fALS cases with known mutations.

*Benefits and Limitations of Antisense Oligonucleotides*​​​​​​​ *(ASOs) for Amyotrophic Lateral Sclerosis​​​​​​​ (ALS)*

ASO therapy offers significant benefits, particularly its high specificity in targeting known mutations. This is especially relevant in familial ALS, where a single, well-characterized mutation is typically responsible. Additionally, intrathecal administration enables ASOs to bypass the blood-brain barrier and reach the central nervous system effectively [12,13].

However, limitations remain. Familial ALS accounts for only about 10% of cases [13], so most patients with sporadic ALS may not benefit from current ASO designs. Furthermore, ASOs cannot reverse existing neurodegeneration and depend on early intervention. Unfortunately, by the time symptoms are clinically evident, irreversible motor neuron damage has often already occurred.

Therefore, while ASOs represent a promising therapeutic breakthrough, their effectiveness is currently limited by the need for early diagnosis and the complex genetic landscape of ALS.

Mechanisms and Experimental Results for Common Causative Genes

Different gene and clinical trial descriptions: Antisense oligonucleotide (ASO) therapies for ALS have primarily targeted genes strongly associated with familial ALS (fALS). These include SOD1, C9ORF72, FUS, TARDBP, and ATXN2. Below is a synthesis of their functions, mutation-related pathologies, and current ASO trial results-framed with attention to broader therapeutic implications (Table 2).

**Table 2:** Experimental results for common ALS causative genes. ALS: amyotrophic lateral sclerosis, FUS: fused in sarcoma, ATXN2: Ataxin-2, SOD1: silence superoxide dismutase 1. Table credits: Edward Jeong.

ASO	Target gene	Trial phase	Outcome	Status
Tofersen [12]	SOD1	Phase III	FDA-approved	Approved
BIIB078 [11]	C9ORF72	Phase I	No efficacy endpoints met	Discontinued
WVE-004 [11]	C9ORF72	Phase I	No efficacy endpoints met	Discontinued
Afinersen [11]	C9ORF72	Single patient, no clinical trial	Ongoing	Ongoing
ION363 (Jacifusen) [11]	FUS	Single patient, Phase III	Some biomarker response	Ongoing
BIIB105 [11]	ATXN2	Phase I	No clinical improvement	Discontinued

Superoxide dismutase type 1 (SOD1): The SOD1 gene encodes a mitochondrial enzyme that helps neutralize oxidative stress and facilitates protein turnover [9,14]. Mutations in SOD1 lead to protein misfolding and aggregation, which in turn contribute to toxic oligomer formation and motor neuron death-making SOD1 a key ASO target. Tofersen, an ASO designed to silence SOD1, has been the most successful therapy to date. Administered intrathecally to bypass the blood-brain barrier [15], Tofersen has demonstrated a dose-dependent reduction of cerebrospinal fluid (CSF) SOD1 protein levels by up to 36% in early trials [6]. The VALOR Phase III trial confirmed reductions of 29-40% across patient subgroups [12,16-18], and Tofersen received FDA approval in 2023 as the first ASO officially sanctioned for ALS treatment. 

Chromosome 9 open reading frame 72 (C9ORF72): C9ORF72 is involved in autophagy, endosomal trafficking, and RNA splicing [9]. A hexanucleotide repeat expansion in this gene (G4C2) is the most common genetic cause of familial ALS, particularly in European populations [19]. These repeats impair variant 1 expression and disrupt cellular homeostasis.

Three ASO therapies have targeted C9ORF72: BIIB078: A Phase I trial involving 114 patients reported no significant clinical improvements, despite a favorable safety profile. The trial was discontinued due to lack of efficacy [11]. WVE-004: Assessed in the FOCUS-C9 Phase Ib/IIa trial, this ASO also failed to meet efficacy endpoints and was discontinued [11,20,21]. Afinersen: Administered on a compassionate-use basis to a single patient, this ASO showed minimal clinical benefit and remains under informal evaluation. These trials underscore the difficulty of translating molecular rationale into clinical benefit, especially when targeting complex repeat expansions.

Fused in sarcoma (FUS): FUS encodes an RNA-binding protein essential for RNA splicing, DNA repair, and stress granule formation [9]. ALS-associated mutations often result in cytoplasmic mislocalization of FUS, leading to axonal dysfunction and early-onset ALS, particularly in patients under 40 [11,22,23].

ION363 (Jacifusen) is an ASO that targets the C-terminal region of mutant FUS transcripts. It was first administered to a 26-year-old patient with advanced ALS under an expanded access protocol. Although the patient died within months, post-treatment analysis showed reductions in mutant FUS protein levels and aggregation [11,24]. Encouraged by these findings, a Phase III trial of Jacifusen is now ongoing. This case illustrates the potential for personalized ASO design, especially when administered early in the disease course.

TAR DNA Binding Protein (TARDBP) and Ataxin-2 (ATXN2): TARDBP: TARDBP encodes the TDP-43 protein, which is critical for RNA processing and miRNA regulation [9]. Over 50 mutations have been linked to ALS, with disease mechanisms driven by TDP-43 misfolding and aggregation. Certain mutations (e.g., D169G, R361S) are associated with stress granule dysfunction and disrupted protein interactions, particularly with FUS and ATXN2.

Although TARDBP is a key pathogenic driver in both familial and sporadic ALS, no ASOs targeting this gene have progressed beyond preclinical studies. Its role remains a priority for future ASO development, though its intricate interactions pose challenges for therapeutic design.

ATXN2: ATXN2 is involved in stress granule regulation and modulates TDP-43 toxicity. Intermediate-length polyglutamine (polyQ) expansions in this gene have been shown to exacerbate TDP-43 aggregation and increase neuronal vulnerability [11,25].

BIIB105, an ASO targeting ATXN2, entered a Phase I trial in 2020. However, due to limited observed clinical improvement, the trial was discontinued [11]. This reflects the difficulty in targeting modifier genes where causality is indirect and therapeutic thresholds are less clear.

Summary of Therapeutic Trends

Across these trials, a few consistent themes emerge. While SOD1-targeted therapy has shown clear clinical and biomarker-based efficacy, ASOs aimed at C9ORF72, FUS, and ATXN2 have encountered barriers-ranging from insufficient clinical endpoints to challenges in trial design and delivery timing. The majority of failed trials did not show adverse effects, suggesting safety is not the primary hurdle, but rather achieving meaningful functional outcomes.

As ASO technology advances, lessons from these early trials may inform more precise, combinatory, and biomarker-guided approaches. These will be essential for addressing the genetic complexity of ALS, especially in sporadic forms where multi-targeted strategies may become necessary.

Discussion

Among the six ASO programs reviewed, only Tofersen has demonstrated sufficient clinical and biomarker-based efficacy to earn FDA approval. Most other ASO trials showed favorable safety outcomes but failed to meet efficacy endpoints, highlighting the inherent challenges in treating a complex, polygenic disease like ALS.

The studies reviewed varied in their methodological rigor and risk of bias. For instance, single-patient trials, such as Jacifusen and Afinersen, lacked control groups and blinding, which limits the generalizability of their results and may inflate therapeutic expectations. Early-phase studies, such as BIIB105, frequently suffered from incomplete or unclear reporting, making it difficult to fully assess their internal validity. Despite these limitations, each study contributes valuable insights that lay the groundwork for future innovations in the field.

Emerging gene-editing technologies such as Clustered Regularly Interspaced Short Palindromic Repeats (CRISPR) offer the potential for permanent gene correction, but they remain in early development stages and face their own ethical and technical hurdles. For now, ASOs remain the most advanced and customizable genetic therapy platform, especially for monogenic forms of ALS.

However, the clinical landscape of ALS is dominated by sporadic ALS, which accounts for approximately 90-95% of cases. These cases often involve multiple, low-effect mutations or epigenetic factors, making them unsuitable for single-gene-targeted therapies. Future ASO development must evolve toward multi-targeted or combinatory approaches, supported by biomarker-guided precision trials to identify patients who may benefit from individualized treatment regimens.

In complex cases, combining ASOs with other therapeutic agents, such as neuroprotective drugs, anti-inflammatory agents, or RNA-targeting molecules, may enhance efficacy. Additionally, earlier diagnostic tools and faster genetic profiling could help initiate treatment at a stage when motor neurons are still salvageable.

Importantly, failed ASO trials should not be viewed as setbacks but as stepping stones that enrich our understanding of ALS pathophysiology and therapeutic thresholds. Even when clinical benefits were not observed, these trials helped refine delivery methods, improve safety monitoring, and identify biomarkers for future study. Each iteration brings the field closer to gene-specific, effective interventions that can meaningfully alter disease progression.

## Conclusions

This umbrella review concludes that while antisense oligonucleotides (ASOs), particularly Tofersen, represent a significant advancement in the treatment of genetically defined ALS, their clinical utility remains limited by current diagnostic, delivery, and genetic targeting constraints. Of the six prominent ASO therapies reviewed, only one has received FDA approval. Three others were discontinued due to a lack of clinical benefit, highlighting the high attrition rate in ASO development. These setbacks, however, underscore the need for continued research into more effective delivery mechanisms, earlier diagnostics, and more adaptive trial designs. While the development of ASOs for ALS has not been linear or easy, it has steadily advanced through iterative learning, trial refinement, and enhanced biological understanding.

With sustained investment in translational research, patient stratification, and precision medicine, ASOs hold the potential to expand beyond a niche application for familial ALS. They may eventually become a versatile therapeutic option for a broader population, including those with sporadic ALS and other neurodegenerative diseases. In summary, the future of ASO therapy for ALS lies not only in improving individual drugs but in building a comprehensive, multi-faceted treatment framework grounded in genetic precision, timely intervention, and clinical adaptability. Expanding research in this area is both necessary and promising.
